# Supplementary material for: Maximizing Glycoproteomics Results through an Integrated Parallel Accumulation Serial Fragmentation Workflow
Source: Anal Chem. 2024 May 22;96(22):8956–64. doi: 10.1021/acs.analchem.3c05874 (PMC11154686; doi:10.1021/acs.analchem.3c05874)
Supplement: Supplementary file 1 — ac3c05874_si_001.pdf [file ac3c05874_si_001.pdf]

Supporting Information

Maximizing glycoproteomics results through an integrated PASEF workflow

Melissa Baerenfaenger[1, 2†], Merel A Post[1†], Fokje Zijlstra[3], Alain J van Gool[3], Dirk J Lefeber[1, 3], Hans JCT Wessels[3*]

1. Department of Neurology, Donders Institute for Brain, Cognition, and Behavior, Radboud University Medical Center, Nijmegen, Netherlands
2. Division of BioAnalytical Chemistry, AIMMS Amsterdam Institute of Molecular and Life Sciences, Vrije Universiteit Amsterdam, Amsterdam, Netherlands
3. Translational Metabolic Laboratory, Department of Human Genetics, Radboud University Medical Center, Nijmegen, Netherlands

†These authors contributed equally to this work and share first authorship

*Correspondence: Hans.Wessels@radboudumc.nl

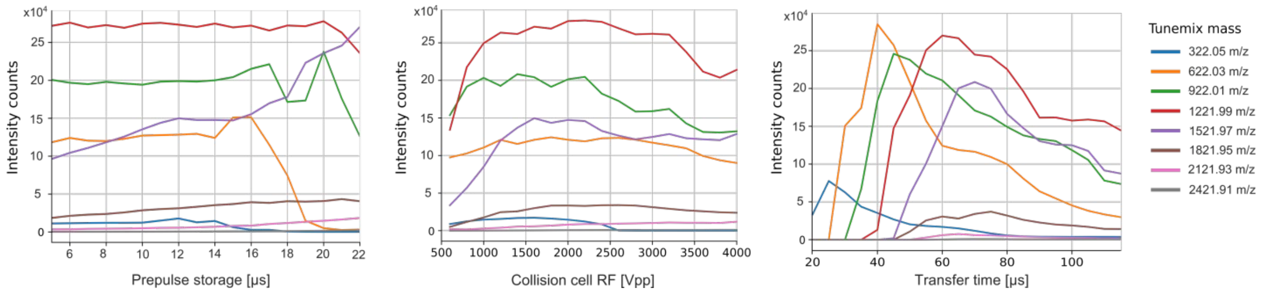

Figure S1. Ion optics tuning with direct infusion of tunemix (Agilent Technologies) using the proteomics method. The intensity of different tunemix ions is plotted against the prepulse storage, collision cell RF and transfer time. Low mass ions, like 322.05 m/z, benefit from short prepulse storage, low collision cell RF and short transfer times, whereas higher mass ions benefit from longer prepulse storage, higher collision cell RF and shorter transfer times. For ideal detection of glycopeptides and glycopeptide fragments, low and high mass ions need to be detected to ensure detection of high mass glycopeptide precursors and low mass oxonium ions.

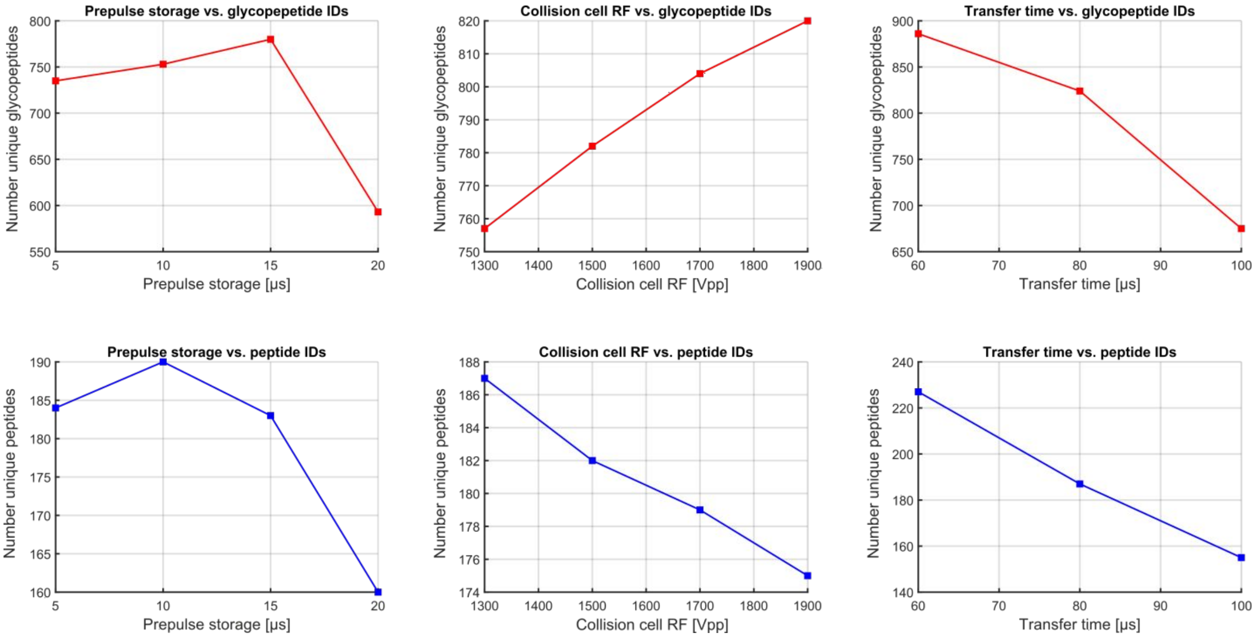

Figure S2. Tuning of ion optics for ideal transmission and identification of glycopeptides via optimization of prepulse storage time, collision cell RF and transfer time. Shown in top panels are glycopeptide IDs (red) whereas the bottom panels show peptide IDs (blue). Of particular interest is the collision cell RF. A higher collision cell RF is favorable for glycopeptide identifications. This is potentially due to the occurrence of glycopeptide fragment ions at higher m/z values caused by fragmentation of the glycan moiety with intact peptide moiety. These fragment ions, in particular the peptide+HexNAc peak, are relevant for the correct assignment of the peptide mass of glycopeptides and benefit from higher collision cell RF values. Although 1900 Vpp shows the highest number of glycopeptide IDs, we selected a value of 1700 Vpp for the following measurements since settings above 1700 Vpp led to a significant signal loss of low m/z oxonium ion intensities. The detection of oxonium ions at low m/z values can be critical for other data interpretation tools.

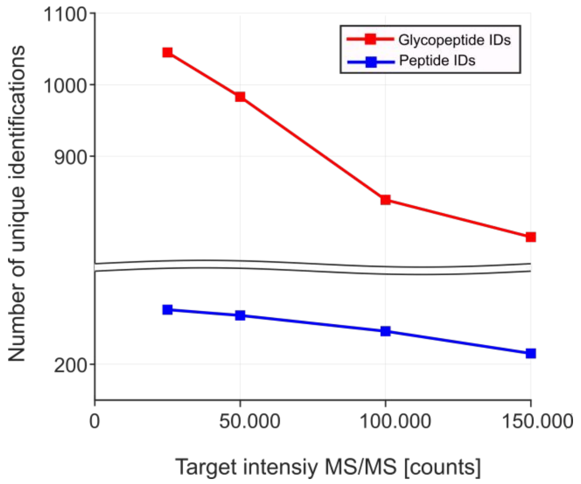

Figure S3. Optimization of target intensity. Both for peptides and glycopeptides, increasing the target intensity does not improve identification. For the developed glycoproteomics method, a target intensity of 25.000 counts was selected which is comparable to the target intensity of 20.000 in the default proteomics method.

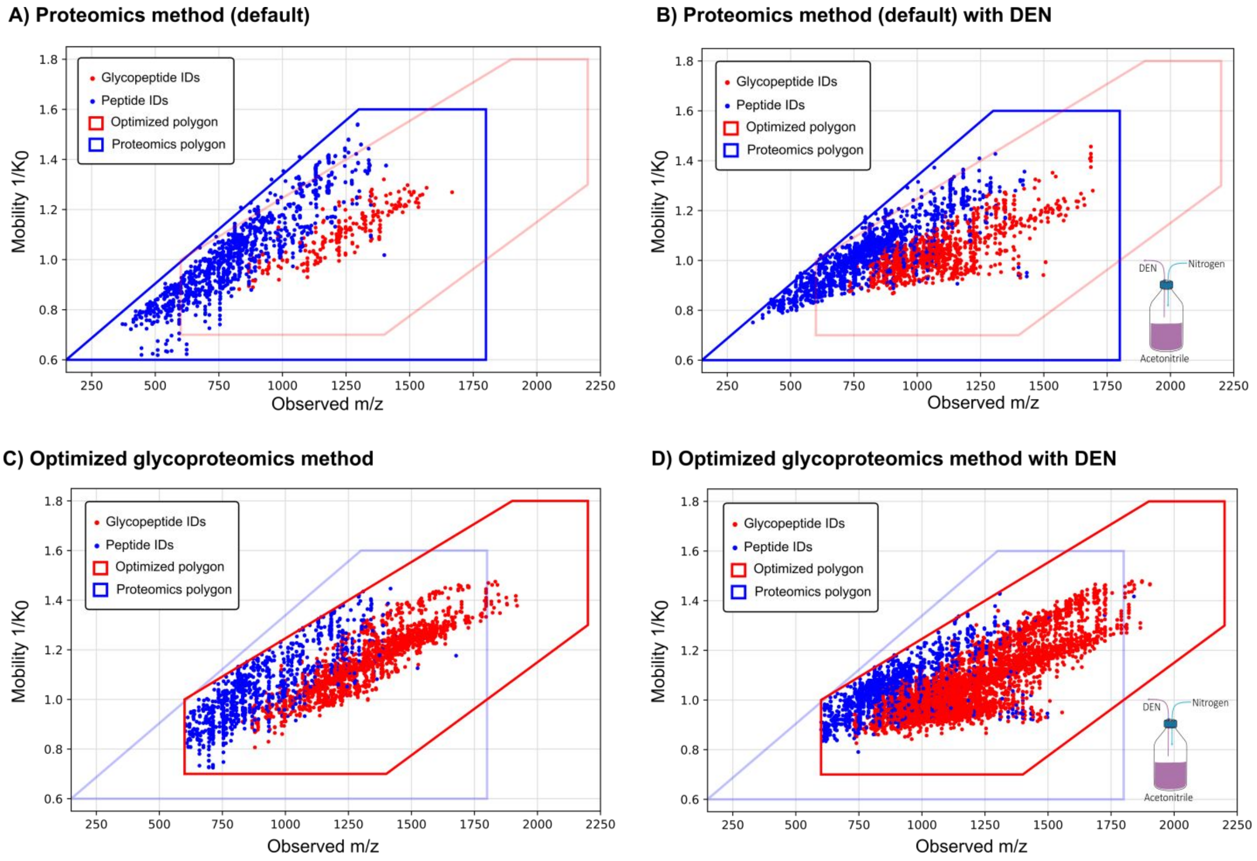

Figure S4. Glycopeptide and peptide IDs plotted by m/z vs. mobility value for proteomics method and optimized glycoproteomics method with and without the use of dopant enriched nitrogen gas via nanobooster. The default polygon used in the proteomics method is depicted in blue and the optimized polygon for glycopeptide identification is plotted in red. The proteomics polygon was used in A and B, the glycoproteomics polygon was used in C and D.

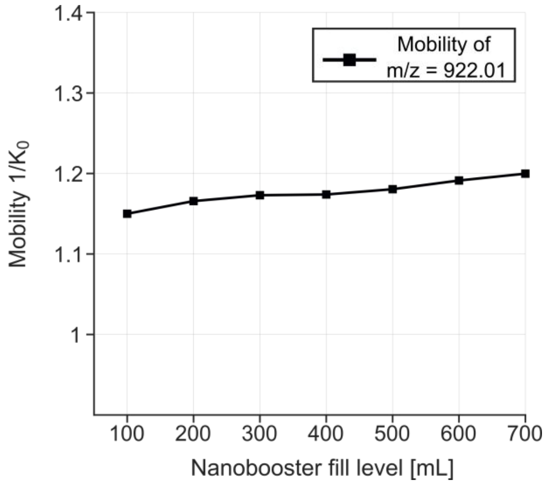

Figure S5. Change in mobility of one agilent tunemix (Agilent Technologies) signal at m/z = 922.01 with increasing fill levels of the nanobooster with acetonitrile. When using DEN, the mobility of the ions shifts as the collision gas density, flow rate and possibly temperature change. In addition, there is a slight shift in mobility over time as the solvent content in the nanoBooster decreases as shown here. This mobility shift can be well controlled if the nanoBooster is refilled regularly.

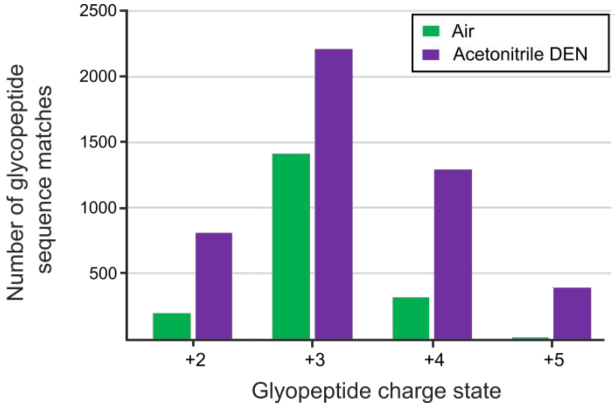

Figure S6. Charge state distribution of glycopeptides with and without acetonitrile-enriched nitrogen gas using the optimized glycoproteomics method. The number of glycopeptide sequence matches represents the average between two replicate measurements. A dominant charge state of +3 is observed without the use of DEN. When using DEN, the chare state distribution shift towards an almost equal amount of +3 and +4 precursor ions.

Table S1. Tested nanoLC gradients. Solvent A was composed of water with 0.1% FA and 0.02% TFA. Solvent B was composed of acetonitrile with 0.1% FA and 0.02% TFA. The column was operated at 45 °C.

Solvent B in %	Gradient time:				
	**5 min**	**15 min**	**30 min**	**45 min**	**60 min**
1	0 min	0 min	0 min	0 min	0 min
7	1 min	1 min	1 min	1 min	1 min
45	6 min	16 min	31 min	46 min	61 min
90	7 min	17 min	32 min	47 min	62 min
90	9 min	19 min	34 min	49 min	64 min

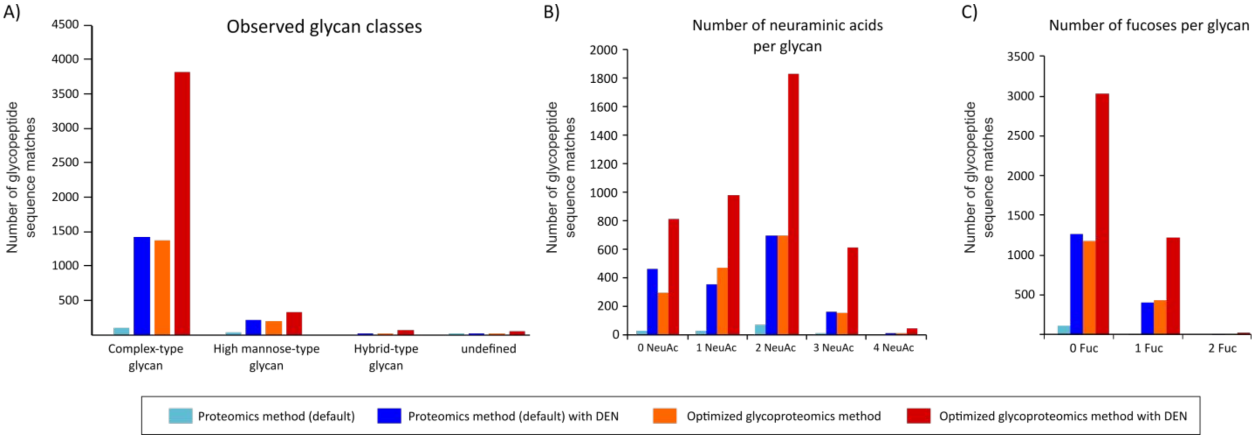

Figure S7. Grouped glycopeptide sequence matches for the default proteomics method, the proteomics method with the use of DEN, the optimized glycoproteomics method and the optimized glycoproteomics method with use of DEN. The number of glycopeptide sequence matches are the sum of two replicate measurements. A) For all methods, complex-type glycans are the dominant glycan class, followed by high-mannose type. B) The number of neuraminic acids per glycan compositions. Highly sialylated glycans (3 and 4 neuraminic acids) were dominantly detected with the optimized glycoproteomics method using DEN. C) The number of fucose units per glycan composition.

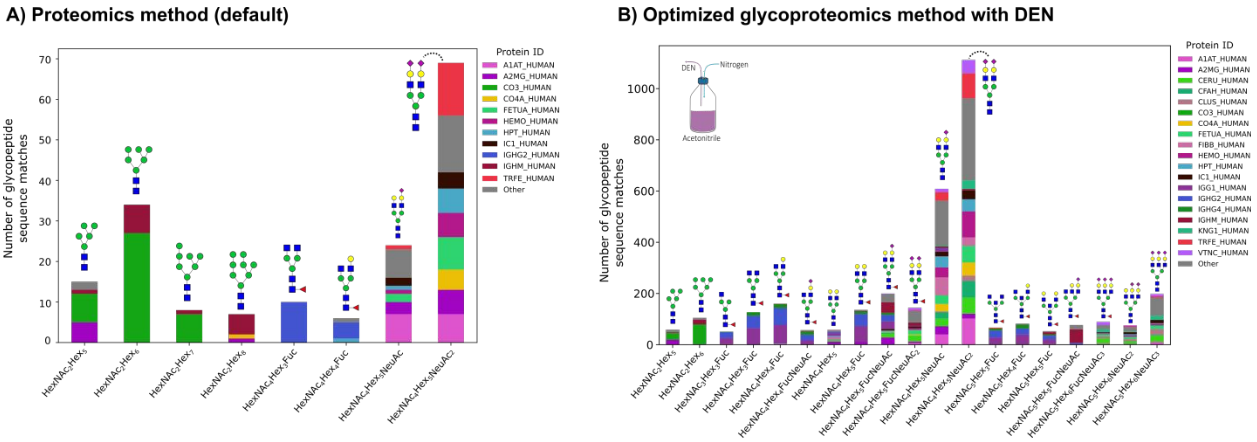

Figure S8. Identified glycopeptide sequence matches for proteomics method and optimized glycoproteomics method with the use of DEN for one selected measurement. (A) The proteomics method resulted in the identification of glycopeptides from 26 different glycoproteins. (B) Glycopeptides from 80 different glycoproteins were identified with the optimized glycoproteomics method and the use of DEN. The figure only shows glycan composition with a high occurrence (A: over 5 glycopeptide sequence matches, B: over 50 glycopeptide sequence matches). A table of all identifications for all testes parameters can be found in the ProteomeXchange repository under the identifier PXD047898. It is worth noting that the number of glycopeptide sequence matches does not represent quantitative information but only qualitative annotations. The annotation with glycan structures represents only one possibility. Other isomers are plausible.

Table S2. Settings of the default proteomics method and optimized parameters for the developed glycoproteomics method.

**Parameter**	**Proteomics method**	**Optimized glycoproteomics method**
Collision energies [eV]	0.6 $1/K_0$ = 20 eV<br>1.6 $1/K_0$ = 59 eV	0.6 $1/K_0$ = 30 eV<br>1.6 $1/K_0$ = 89 eV
Collision cell RF [Vpp]	1500	1700
Prepulse storage [µs]	12	10
Transfer time [µs]	60	60
Target intensity PASEF [counts]	20.000	25.000
TIMS isolation width [ms]	2.75	7.50
Polygon coordinates for precursor selection	150 m/z @ 0.6 $1/K_0$,<br>1300 m/z @ 1.6 $1/K_0$,<br>1800 m/z @ 1.6 $1/K_0$,<br>1800 m/z @ 0.6 $1/K_0$	600 m/z @ 1.0 $1/K_0$,<br>1900 m/z @ 1.8 $1/K_0$,<br>2200 m/z @ 1.8 $1/K_0$,<br>2200 m/z @ 1.3 $1/K_0$,<br>1400m/z @ 0.7 $1/K_0$,<br>600 m/z @ 0.7 $1/K_0$
Mass range [m/z]	100-1700	50-4000
Mobility range [$1/K_0$]	0.6-1.6	0.7-1.5
Delta potentials [V]	D1: -20<br>D2: -160<br>D3: 110<br>D4: 110<br>D5: 0<br>D6: 55	D1: -20<br>D2: -160<br>D3: 110<br>D4: 110<br>D5: 0<br>D6: 55
Ramp time [ms]	100	100
Accumulation time [ms]	100	100

Table S3. Optimization scheme.

Optimization step	Nanobooster fill level (ACN) [mL]	CE energy	Collision cell RF [Vpp]	Prepulse storage [µs]	Transfer time [µs]	Target intensity PASEF	Tims isolation width [ms]	Polygon	Gradient length [min]	Mobility range [1/K_0]
CE energies	600	x	1500	12	100	100 K	2.75	Default	15	0.6-1.6
Collision cell RF [Vpp]	600	+50 %	x	12	80	100 K	2.75	Default	15	0.6-1.6
Prepulse storage [µs]	600	+50%	1500	x	80	100 K	2.75	Default	15	0.6-1.6
Transfer time [µs]	600	+50%	1500	12	x	100 K	2.75	Default	15	0.6-1.6
Target intensity PASEF	600	+50%	1700	10	80	x	2.75	Default	15	0.7-1.5
Tims isolation width [ms]	600	+50%	1700	10	60	25 K	x	Default	15	0.7-1.5
Polygon	600	+50%	1700	10	60	25 K	7.5	X	15	0.7-1.5
Gradient length [min]	600	+50%	1700	10	60	25 K	7.5	Optimized	x	0.7-1.5
Benchmarking										
Proteomics method (default)	No DEN (Air)	+ 0 %	1500	12	60	20 K	2.75	Default	30	0.6 – 1.6
Proteomics method (default) + DEN	600	+ 0 %	1500	12	60	20 K	2.75	Default	30	0.6 – 1.6
Glycoproteomics methods	No DEN (Air)	+ 50 %	1700	10	60	25 K	7.5	Optimized	30	0.7 – 1.5
Glycoproteomics methods + DEN	600	+ 50 %	1700	10	60	25 K	7.5	Optimized	30	0.7 – 1.5
